# Hierarchical Fibroid Vision Transformer (HiFi-ViT): A Hierarchical Vision Transformer Framework for Accurate Uterine Fibroid Detection in Ultrasound Imaging

**DOI:** 10.7759/cureus.105197

**Published:** 2026-03-13

**Authors:** Precious Ogie, Pratibha Srikanthan, Gbadegesin Taiwo, Olumide Ogundepo

**Affiliations:** 1 General Medicine, University Hospitals of North Midlands, Stoke-on-Trent, GBR; 2 General Medicine, Lewisham and Greenwich NHS Trust, London, GBR; 3 Artificial Intelligence, University of Salford, Salford, GBR; 4 Statistics, University of Ilorin, Ilorin, NGA

**Keywords:** artificial intelligence, deep learning, fibroid detection, hierarchical vision transformer, medical imaging, ultrasound imaging, uterine fibroids, vision transformer

## Abstract

Uterine fibroids are common benign tumors that originate from the smooth muscle layer of the uterus and are often associated with reproductive complications. Early identification is important for timely management and the prevention of complications such as infertility. However, diagnosing fibroids can be challenging because their symptoms often overlap with other gynecological conditions, such as adenomyosis and ovarian cysts, which may lead to delayed diagnosis or misdiagnosis.

In this study, we propose a two-stage hierarchical deep learning framework that combines a Detection Transformer (DETR) for fibroid localization with an ensemble of Vision Transformers for classification using ultrasound images. The dataset was first divided at the image level prior to patch extraction, after which fibroid and normal tissue patches were generated from each subset to support model training and evaluation. This two-stage approach enables improved localization while addressing class imbalance and preserving relevant contextual information in ultrasound images.

The proposed framework achieved high performance on the available dataset, with an accuracy of 99.42%, a precision of 99.10%, a recall of 99.36%, an F1 score of 99.42%, and an area under the curve (AUC) of 99.9%. However, the dataset contains a limited number of normal cases and does not include patient-level identifiers, which may affect the generalizability of the results. Therefore, further validation on larger and more diverse clinical datasets is necessary before clinical deployment.

## Introduction

Fibroids are benign or non-cancerous growths that develop in a woman's uterine muscular wall. The prevalence of these uterine fibroids exceeds 50% in women by age 45 [1], and they are typically detected in middle-aged and older women. Common signs of uterine fibroids include heavy menstrual bleeding, pelvic discomfort, and infertility [1]. The precise cause of uterine fibroids is unknown, although genetics and hormone abnormalities may be contributing factors. Up to 80% of women are expected to develop uterine fibroids by the age of 50, making them one of the most common health issues for women [2]. Notably, research suggests that women of African heritage are up to three times more at risk [3]. This high prevalence underscores the clinical importance of timely and accurate detection of uterine fibroids. In addition to their negative effects on an individual's health (such as pain, infertility, and pregnancy-related dangers), fibroids also have a significant financial cost.

It is estimated that the annual cost of managing fibroids, including treatments and fertility services, as well as lost productivity, exceeds USD 42 billion [4]. The fibroid size, type, location, and degeneration can all vary greatly [5]. Because of this diversity, a one-size-fits-all strategy is not always successful; instead, treatment needs to be carefully customized based on variables such as the number of fibroids and their features, as well as symptoms, age, and fertility objectives [6]. Because uterine fibroids are a leading cause of hysterectomy worldwide, they have a substantial impact on women's reproductive health and quality of life [7]. A common non-invasive method for diagnosing and tracking uterine fibroids is ultrasound imaging. Medication is typically recommended to treat the symptoms and minimize the growth of fibroids [8]. However, it can be difficult to manually identify uterine fibroids in the ultrasound for minor or concealed lesions. Many existing deep learning approaches focus primarily on classification without explicitly incorporating a detection stage to localize fibroid regions within ultrasound images. To address this limitation, this study proposes Hierarchical Fibroid Vision Transformer (HiFi-ViT), a two-stage hierarchical framework that combines a Detection Transformer (DETR) for fibroid localization with an ensemble of Vision Transformers (ViT) for robust classification.

As a result, deep learning presents a viable technique for automatically classifying uterine fibroids in ultrasound pictures. According to the literature study, deep learning models have demonstrated promising performance in distinguishing fibroids from non-fibroid uterine conditions in ultrasound imaging [9]. Additionally, it helped in the elimination of subjective and human bias from classification [9], which produced consistent results. Automated classification may result in the early identification and detection of uterine fibroids, enabling quick intervention and offering better patient results by saving radiologists' time for other more important tasks. Therefore, in this proposed research, DETR [10] is combined with an ensemble of ViT [11] for ultrasound image uterine fibroid classification. The processes involved in the classification are as follows:

The DETR algorithm was trained to identify the location of fibroids within the ultrasound image data and output bounding boxes. It includes the preprocessing, annotations of XML, and model training as well as validation. 

After training the detection model, it then extracts patches from the dataset. The fibroid class patches are obtained from the detected bounding boxes, while the normal class patches are extracted from regions without fibroids, hence creating a balanced dataset for the next stage. 

An ensemble of ViT that consists of ViT-Base, ViT-Small, and DeiT-Base ViT was trained to classify these extracted patches as either fibroid or normal. The aim of the ensemble is to enhance classification performance. 

The detection and classification system was then evaluated on a test set with reporting metrics like accuracy, receiver operating characteristic (ROC) area under the curve (AUC), precision, recall, specificity, and F1 score. Also, visuals such as architecture diagrams, confusion matrices, ROC curve, and prediction grid were generated for model behavior and performance insight.

Several researchers had worked on uterine fibroid classification. Using a dataset of 3D ultrasound pictures, they employed a 3D convolutional neural network (CNN) and achieved an accuracy of 91.3% [9]. Due to its improved ability to capture spatial information, the researchers praised the 3D CNN points of interest over traditional 2D in identifying uterine fibroids. Using a pre-trained ResNet-50 CNN calibrated on their dataset of 2D ultrasound pictures, Stoelinga et al. [12] achieved 98.8% accuracy.

The suggested model used a collection of convolutional layers to extract highlights from ultrasound pictures. With an accuracy of 97.5%, the suggested model demonstrated the suitability of deep convolutional neural networks (DCNNs) for uterine fibroid assurance [11]. Examiners suggested a cross-breed deep learning [13] depicted to identify uterine fibroids in research [14]. Highlights were eliminated using a combination of CNN and gloomy neural frameworks once the ultrasound images were given into the program. Given that the suggested demonstration achieved an accuracy of 96.8%, the study's findings indicate that the hybrid deep learning has the potential to be used in beneficial image processing. Behboodi et al. [15] suggested another idea through a DCNN approach for the localization of uterine fibroids from ultrasound images. The suggested method uses a combination of convolutional and pooling layers to extract features from ultrasound pictures. With an accuracy of 96.7% [15], the suggested demonstration demonstrated that DCNN can be lucrative for identifying uterine fibroids. In order to extract characteristics from the ultrasounds, a deep learning-based approach for programmed uterine fibroid location from ultrasound images was suggested [16].

## Materials and methods

This study utilized a publicly available dataset of uterine fibroid ultrasound images [17]; it consists of 871 images with annotations. Three thousand four hundred and seventy-three image patches were generated, which consist of 863 as fibroid and 2610 as normal. The train set consists of 2432 patches with 605 patches for the fibroid class and 1827 patches for the normal class, and the validation set consists of 520 patches with 129 patches for the fibroid class and 391 patches for the normal class, while the total patches for the test set consist of 521 patches with 129 patches for the fibroid class and 392 patches for the normal class.

The dataset does not provide patient-level identifiers or metadata indicating whether each image corresponds to a unique patient. Therefore, it cannot be confirmed whether the 871 images represent 871 individual patients or multiple scans from a smaller number of patients. To reduce the risk of direct data leakage, the dataset was first divided at the image level before patch extraction, ensuring that all patches generated from the same image remained within the same subset. However, the absence of patient-level identifiers represents a limitation, as patient-level independence between training and test sets cannot be fully guaranteed.

This section presents and discusses the detection and classification processes from a two-stage hierarchical architecture.

HiFi-ViT

**Figure 1:**
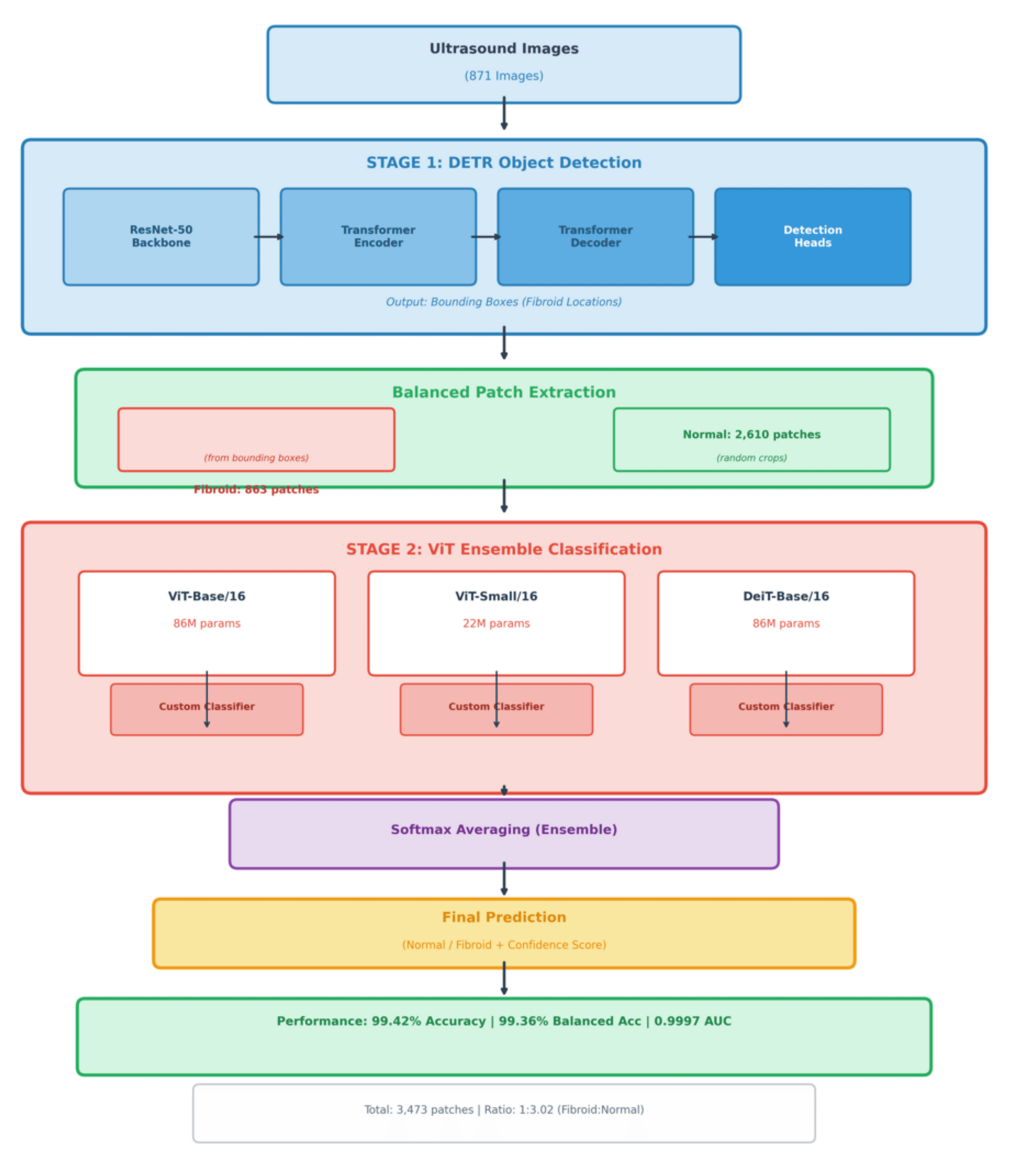
HiFi-ViT two-stage fibroid detection hierarchical architecture overview The HiFi-ViT framework processes 871 ultrasound images through two stages. Stage 1 uses DETR with ResNet-50 [19] backbone to detect fibroids and generate bounding boxes, followed by balanced patch extraction yielding 863 fibroid patches and 2610 normal patches (3473 total, 1:3.02 ratio). Stage 2 employs an ensemble of three ViT [11] (ViT-Base/16 with 86M parameters, ViT-Small/16 with 22M parameters, and DeiT-Base/16 with 86M parameters) combined through SoftMax averaging. The system achieves 99.42% accuracy, 99.36% balanced accuracy, and 0.9997 AUC. HiFi-ViT: Hierarchical Fibroid Vision Transformer; DETR: Detection Transformer; ViT: Vision Transformers; AUC: area under the curve

Figure 1 shows the combined and completed architecture. DETR was selected for its ability to perform end-to-end object localization without anchor-based post-processing, while ViT were chosen for their strength in capturing both local and global contextual information from ultrasound patches. The architecture initializes from input raw ultrasound [18] images and then captures hypoechoic fibroids over myometrial backgrounds with noise. 

Step 1: DETR for Object Detection

The DETR transformer processes raw ultrasound images to output bounding boxes, such that green represents fibroid regions and blue represents normal tissue through an end-to-end prediction without Non-Maximum Suppression (NMS), followed by balanced patch extraction, cropping of fixed-size regions (16×16) from detected regions to develop VI-16 (binary) and DeBI-16 (subtype) datasets, solving the problem of data imbalance. 

Balanced Patch Extraction

The model selects an equal number of normal and fibroid patches after detection and feeds a sample representative into stage 2 while mitigating ultrasound variation such as angles or shadows. 

Stage 2: ViT Classification

Feature extractors: VI-16 and DeBi-16 patches undergo initial embedding such as a CNN transformer integrated for local and global features. 

Aggregator: It is a component responsible for combining multiple patch-level representations into a unified feature descriptor for downstream classification. 

Transformer fusion: It is a phase that applies self-attention to the aggregated patch features in order to integrate contextual relationships between fibroid and normal tissue representations. 

Final prediction: It is the phase where the outcome is classified as either fibroid or normal, with the probability of each class provided alongside the respective confidence score, thereby supporting threshold-based interpretation of the result.

Output metrics

The bottom block shows that validated performance with a near-perfect discrimination suits real-time screening, with hierarchy enabling the powerful handling of cases of small fibroids. 

**Figure 2:**
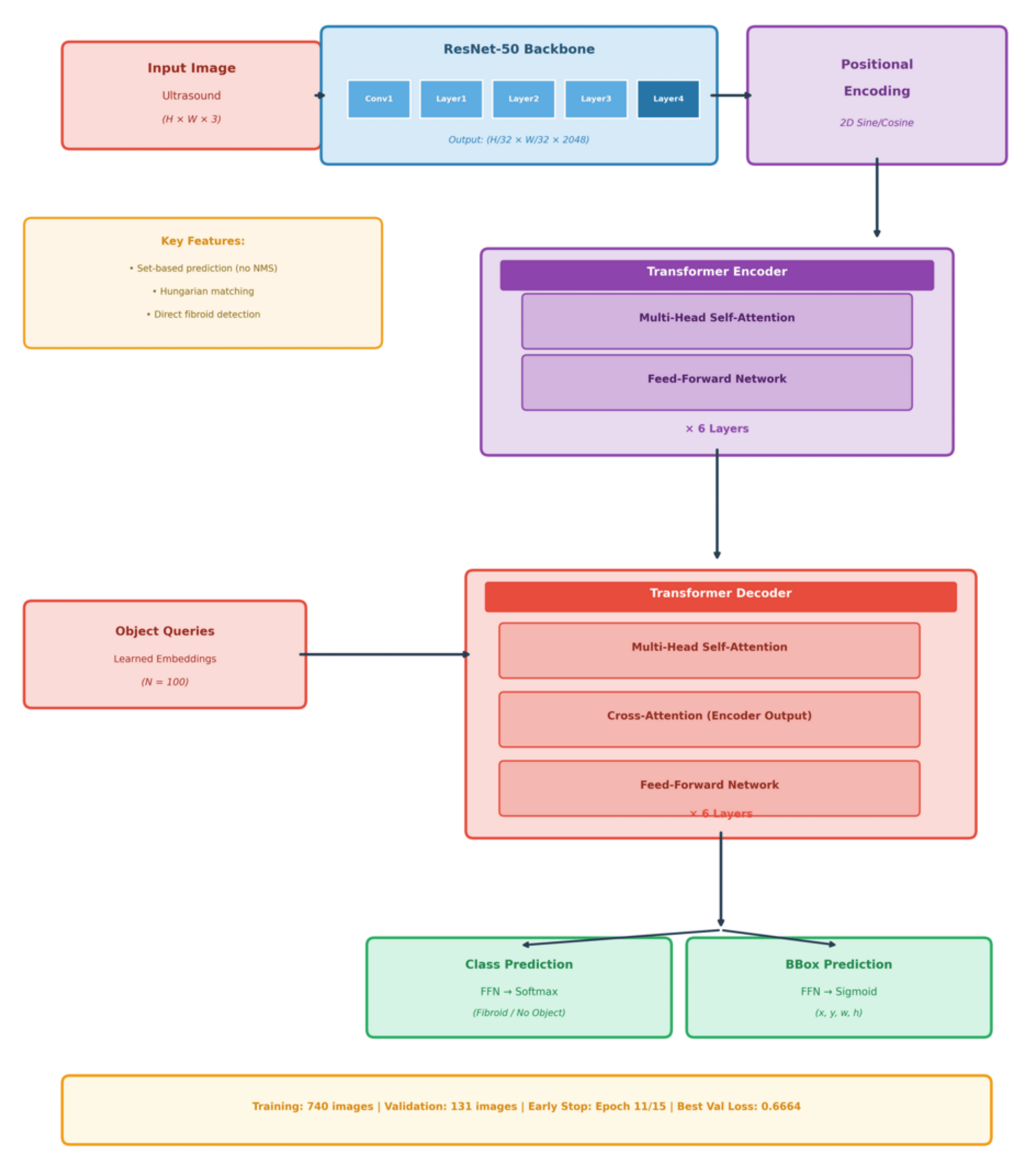
DETR-based fibroid detection architecture The DETR architecture uses a ResNet-50 [10] backbone to extract features from ultrasound images, followed by a six-layer transformer encoder with positional encoding and a six-layer transformer decoder with 100 object queries. Two parallel prediction heads output class labels (fibroid/no object) and bounding box coordinates. The model was trained on 740 images and validated on 131 images, achieving early stopping at epoch 11/15 with validation loss 0.6664. DETR: Detection Transformer

Figure 2 shows a DETR architecture specifically tailored for medical imaging, specifically for detecting fibroids in ultrasound images. Unlike conventional computer vision models that rely on complex hand-crafted components such as anchor boxes or NMS for object detection, DETR [10] handles object detection as a direct prediction problem. Additionally, the global context helps to present the whole image to avoid false positives caused by local artifacts as ultrasound data are too noisy. 

The Backbone (Feature Extraction)

The ResNet-50 [19] CNN was used to start the feature extraction process as it takes the high-resolution ultrasound image data and compresses it into a high-level 2D feature. Also, it reduces the image into a feature map of size 32×32, hence usable for the transformer model while retaining its spatial and informational context that is necessary to locate abnormality. 

The Transformer Core (The Engine)

The engine of this architecture is the Encoder-Decoder transformer structure.

Encoder: It processes the flattened feature map with the multi-head self-attention that helps to look at the model at once and provides global contextual understanding. 

Decoder: This is responsible for looking for images in regions using cross-attention to assign queries to features that are provided by the encoder. 

Parallel Prediction Heads

The DETR predicts all images in a single pass, and the result from the decoder is then fed into the two parallel feed-forward networks that consist of class heads and bounding box heads responsible for class identification (fibroid or normal) in the background and the prediction of exact coordinates for the bounding box, respectively. 

Loss Functions and Training

The architecture consists of several techniques that help optimize predictive accuracy and overall model performance. The Hungarian matching [20], a bipartite matching model that distinctively maps one ground-truth image to one predicted bounding box, helps to eliminate the need for duplicate box removal, solving the anchor or NMS problem.

Losses

The training objectives consist of focal loss [21], which helps to handle class imbalance between small fibroid regions and large background areas, together with L1 loss and GIoU loss [22] to improve the precision of predicted bounding boxes around fibroid regions.

**Figure 3:**
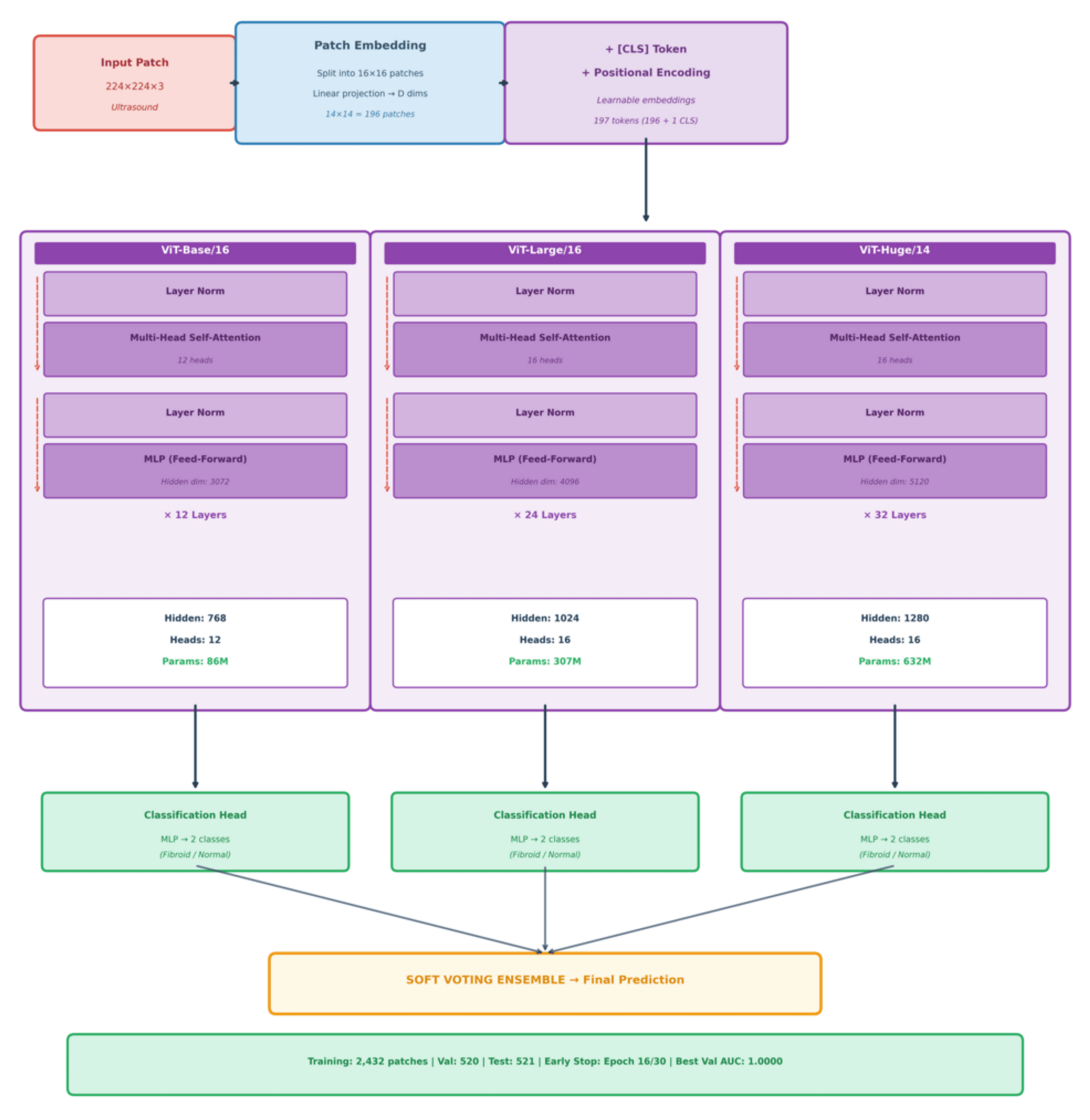
ViT ensemble fibroid classification architecture Input patches (224×224×3) are split into 16×16 patches and linearly projected to create 196 patch embeddings, with a learnable (CLS) token and positional encodings added. Three ViT models process these embeddings: ViT-Base/16 (12 layers, 86M parameters), ViT-Large/16 (24 layers, 307M parameters), and ViT-Huge/14 (32 layers, 632M parameters). Each model's classification head outputs fibroid/normal predictions, which are combined through soft voting to produce the final prediction. The ensemble was trained on 2432 patches and validated on 520 patches, achieving the best validation AUC of 1.0000 at epoch 16/30. ViT: Vision Transformers; AUC: area under the curve

Figure 3 shows the ViT architecture [23], a model that utilizes the transformer scalability approach adopted from natural language processing directly to image recognition tasks. Unlike the conventional CNN that uses local filters, ViT handles the image as a sequence of patches. The following processes are involved in the formation of the architecture. 

Input Processing and Embedding

The process initializes with a standard color image of 224×224×3 as the input patch, and the image is then divided into a grid of 14×14 patches, with each consisting of 16×16 pixels. Thereafter, the patches are flattened and assigned through a linear projection to develop a fixed-size embedding. Also, positional embeddings are applied to retain location information as transformers do not inherently understand the spatial arrangement of the patches, while a learnable classification (CLS) is pre-appended to the sequence to serve as the aggregate for the final output. 

Transformer Encoder Blocks

There are 12 transformer encoder blocks, and each of these blocks has three major components:

Multi-head self-attention: Its purpose is to determine the importance of patches that are related, hence capturing the global relationship over the entire image at the same time. 

Layer norm: The input data is normalized to each layer in this section, and it helps to stabilize training and enhance convergence. 

Multi-layer perceptron with a feed-forward network: It helps to individually process each patch embedding after the attention mechanism has shared information across the sequence. 

Extracting the Global Representation

At the end of the 12-block stack, the algorithm ignores most of the sequence and focuses mainly on the classification (CLS) token. The global representation feature ensures that it summarizes image information into a single vector, which is then typically passed to a standard classification head such as SoftMax to predict the class category.

HiFi-ViT: complete data flow

**Figure 4:**
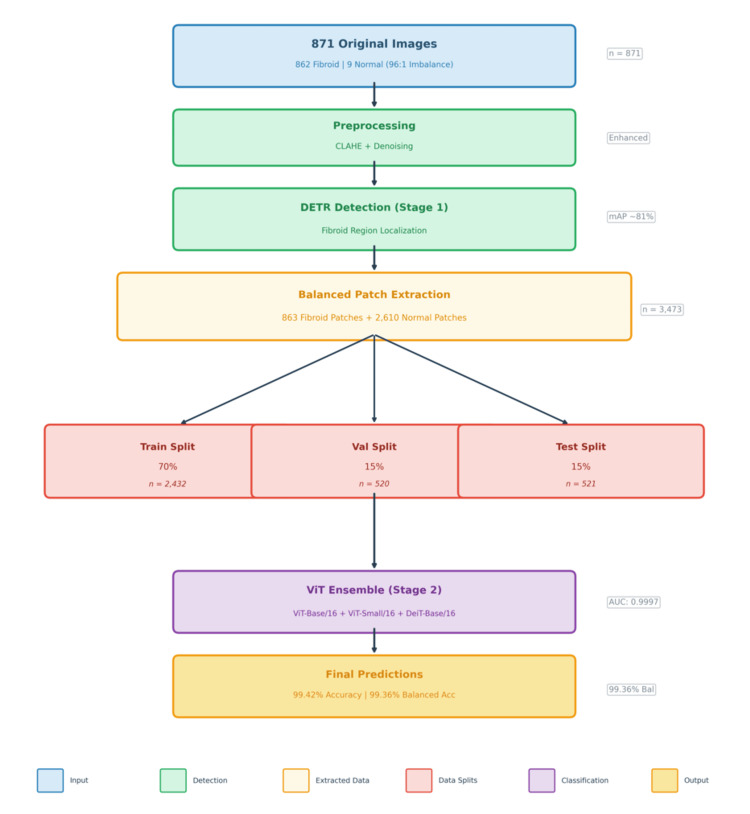
Balanced patch extraction and class imbalance mitigation strategy The workflow begins with 871 original ultrasound images containing 862 fibroid and 9 normal images (96:1 imbalance). After CLAHE and denoising preprocessing, the dataset is first divided at the image level into training (70%), validation (15%), and test (15%) sets. In stage 1, DETR performs fibroid region localization within each subset. Balanced patch extraction then generates fibroid patches from the detected bounding boxes and normal patches from the non-fibroid regions, resulting in 3473 total patches (863 fibroid and 2610 normal). These patches are subsequently used for stage 2 ViT ensemble classification (ViT-Base/16, ViT-Small/16, DeiT-Base/16), achieving the final performance of 99.42% accuracy and 99.36% balanced accuracy. DETR: Detection Transformer; ViT: Vision Transformers; CLAHE: contrast-limited adaptive histogram equalization

The combination of ViT within a larger medical diagnostic framework is referred to as HiFi-ViT in this study. This architecture is designed for high-performance medical diagnosis, majorly for detecting fibroids in ultrasound images (Figure 4). 

This is an end-to-end workflow of the HiFi-ViT system, showing a two-stage process that leads to a final fibroid diagnosis. 

Step 1: Data Preprocessing and Detection

Data preparation: The raw ultrasound data, which consist of 871 images, go through the contrast-limited adaptive histogram equalization (CLAHE) [24] with a denoising approach to ensure the ultrasound details are clearer. 

DETR detection: A DETR responsible for the identification of regions of interest helps to achieve a mean average precision of approximately 81%. 

Patch extraction: This step of the system yields 863 fibroid patches and 2610 normal patches, totaling 3473 extracted data points. 

Step 2: Training and Final Prediction

Data split: The dataset was first divided at the image level into training (70%), validation (15%), and test (15%) sets. Patch extraction was then performed separately within each subset.

The dataset contains a very limited number of original normal ultrasound images (nine images) compared to fibroid images (862 images), resulting in a significant class imbalance.

To mitigate this limitation, additional normal patches were generated through random cropping and data augmentation techniques such as rotation, flipping, color jittering, and CLAHE preprocessing. While this strategy increases the diversity of training samples at the patch level, it does not fully replace true patient-level diversity in healthy uterine tissue. Therefore, this dataset limitation may affect the generalizability of the model to broader clinical populations.

ViT ensemble: The above description of the ViT was used as an ensemble to classify the generated patches. This step achieves an exceptionally high AUC of 0.9997. 

Final results: The architecture concludes with a 99.42% final accuracy and a 99.36% balanced accuracy, showing highly reliable diagnostic performance across both positive (fibroid) and negative (normal) cases. 

Comparison of stages

This study adopted approaches that are different from typical deep learning [25] applications, specifically in the medical domain. The hierarchical two-stage system combines DETR and ViT ensemble for classification. Although there have been several studies that utilize a multi-stage approach, the combination of a transformer-based object detector in an initial stage to localize fibroids, preceded by an ensemble of ViT for the classification of extracted patches, is worthy of an innovative design (Table 1). 

**Table 1:** Diagnostic architecture summary DETR: Detection Transformer; ViT: Vision Transformers; mAP: mean average precision

Feature	Step 1 (DETR)	Step 2 (ViT ensemble)
Primary goal	Detection`	Classification
Input	Improved full images	Extracted patches
Key evaluation metrics	81% mAP	99.42% accuracy

The specialized patch extraction was utilized for balance, considering that there is class imbalance with a total of 3473 patches, that is, 863 for fibroid and 2610 for normal patches. Directly extract fibroid patches from ground-truth bounding boxes and then extract normal patches from random regions such that there is no overlap with any fibroid image. This balanced patch extraction alongside targeting a specific patch per image ratio is critical for solving the class imbalance needed for optimized performance in the second stage. 

The domain-specific ultrasound preprocessing applies a sequence of steps mapped for ultrasound images such as conversion to grayscale, CLAHE for contrast improvement, mean denoising for noise reduction, and a specific crop to focus on the ultrasound region. This approach significantly contributes to the model performance as it prepares the data for optimal use. 

The DETR model initializes with a fibroid label detection, while the background is implicitly handled. An ensemble of different ViT architectures, including ViT-Base, ViT-Small, and DeiT-Base [11], was utilized for final classification, with SoftMax applied to generate class probabilities prior to ensemble prediction. The robustness and higher performance recorded in this research are attributed to ensembled ViT models compared to a single ViT model. 

Finally, a customized collate function was used in training to pad variable-sized images to a common dimension for batching. This was necessary to address the diverse input image sizes that are common in real-world ultrasound data.

## Results

This section presents the results generated from this research with metrics such as accuracy, recall, precision, F1 score, and AUC. Also, the result is presented in visuals like a confusion matrix, ROC curve, precision-recall curve, and visuals on classification of the fibroid and normal images (Table 2).

**Table 2:** Evaluation metrics on test set

Metrics	Score
Accuracy	99.42%
Precision	99.10%
Recall	99.36%
F1 score	99.42%
Sensitivity	99.22%
Specificity	99.49%

Confusion matrix

**Figure 5:**
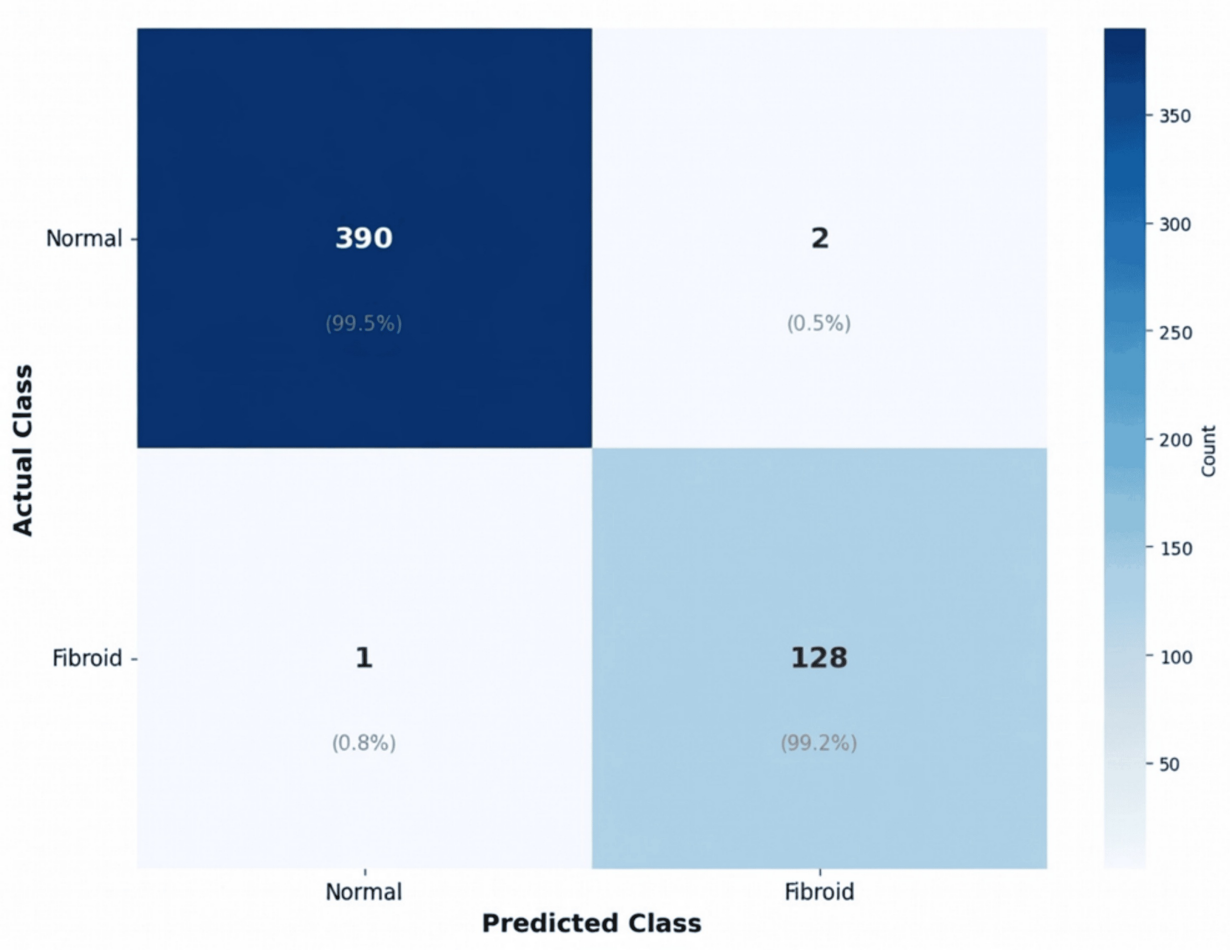
Confusion matrix for uterine fibroid classification The confusion matrix shows HiFi-ViT test set performance on 521 patches (392 normal, 129 fibroid). The model correctly identified 390 normal cases and 128 fibroid cases, with two false positives and one false negative. This corresponds to a specificity of 99.49% (390/392) and a sensitivity of 99.22% (128/129), demonstrating high accuracy in distinguishing fibroid from normal tissue patches. HiFi-ViT: Hierarchical Fibroid Vision Transformer

The confusion matrix provides an explicit breakdown of the algorithm's performance on the test set for the two classes (fibroid and normal). The overall test patch is 521 (392 normal + 129 fibroid) (Figure 5). 

True Positives: 128

These are clinical cases where the model correctly predicted a fibroid when the actual class was fibroid. This shows the model is very good at detecting actual fibroids.

True Negatives: 390

These are clinical cases where the model accurately predicted normal when the actual class was normal. This indicates the model is good at correctly detecting normal issues. 

False Positives: 2

These are clinical cases where the algorithm incorrectly predicted fibroid when the actual class was normal. Only two normal patches were wrongly misclassified as a fibroid. This suggests a very low rate of false alarms, which is crucial in medical diagnosis. 

False Negatives: 1

These are the clinical cases where the model incorrectly predicted normal when the actual class was fibroid. Only one actual fibroid patch was misclassified by the model. This is an exceptionally low rate of misclassified fibroids, showing high sensitivity.

ROC curve

**Figure 6:**
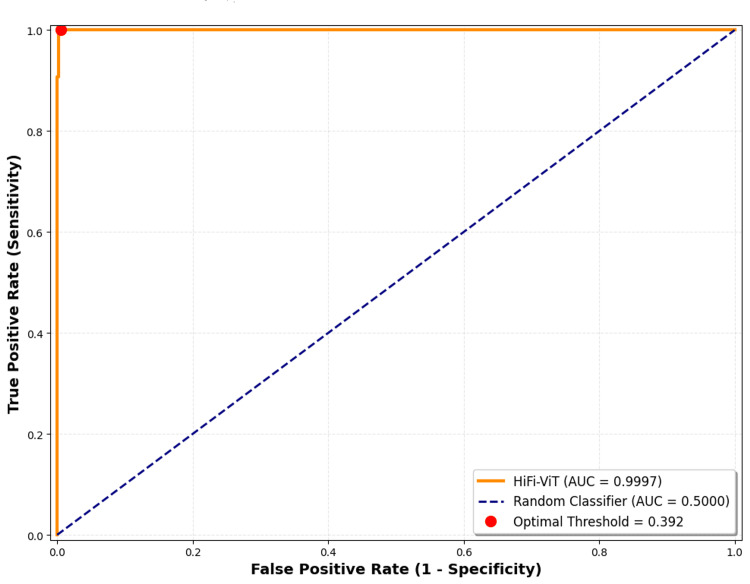
ROC curve analysis for fibroid detection performance The ROC curve demonstrates excellent discrimination ability with an AUC of 0.9997, indicating strong separation between fibroid and normal classes. The point showing 100% sensitivity corresponds to a threshold-dependent analytical operating point derived from ROC analysis and does not represent the final model evaluation used for reporting performance metrics. HiFi-ViT: Hierarchical Fibroid Vision Transformer; ROC: receiver operating characteristic; AUC: area under the curve

Figure 6 shows the ROC curve analysis for fibroid detection performance.

AUC: 0.9997

The AUC score of 0.9997 shows a high and outstanding capacity of the algorithm to distinguish between the normal and fibroid classes. An AUC of 1.0 means a representation of a perfect classifier. Hence, the result of the AUC obtained in this research shows that if a fibroid or normal patch is randomly selected, the algorithm is 99.97% confident that it will allocate a higher probability to the fibroid patch than to the normal patch. 

Optimal Threshold: 0.392

This is the classification threshold that maximizes the difference between the false positive and true positive rate, mostly considered a trade-off between specificity and sensitivity for the given data. If the model's prediction probability for a patch is greater than 0.392, it classifies it as a fibroid, hence classified as normal. 

Sensitivity at Optimal Threshold: 1.000 (100%)

This value corresponds to the theoretical operating point derived from ROC curve analysis at a threshold of 0.392. This threshold was not used for the final evaluation of the model. The actual performance metrics reported in Table 2 were obtained using the standard inference procedure (argmax classification equivalent to a fixed threshold of 0.5), under which the model achieved a sensitivity of 99.22% (128/129) on the test set.

Specificity at Optimal Threshold: 0.9949 (99.49%)

At the optimal threshold, the algorithm accurately identifies 99.49% of actual normal cases. This shows a low false positive rate, such that very few healthy cases are misdiagnosed as positive cases (fibroid). 

Precision-recall curve

**Figure 7:**
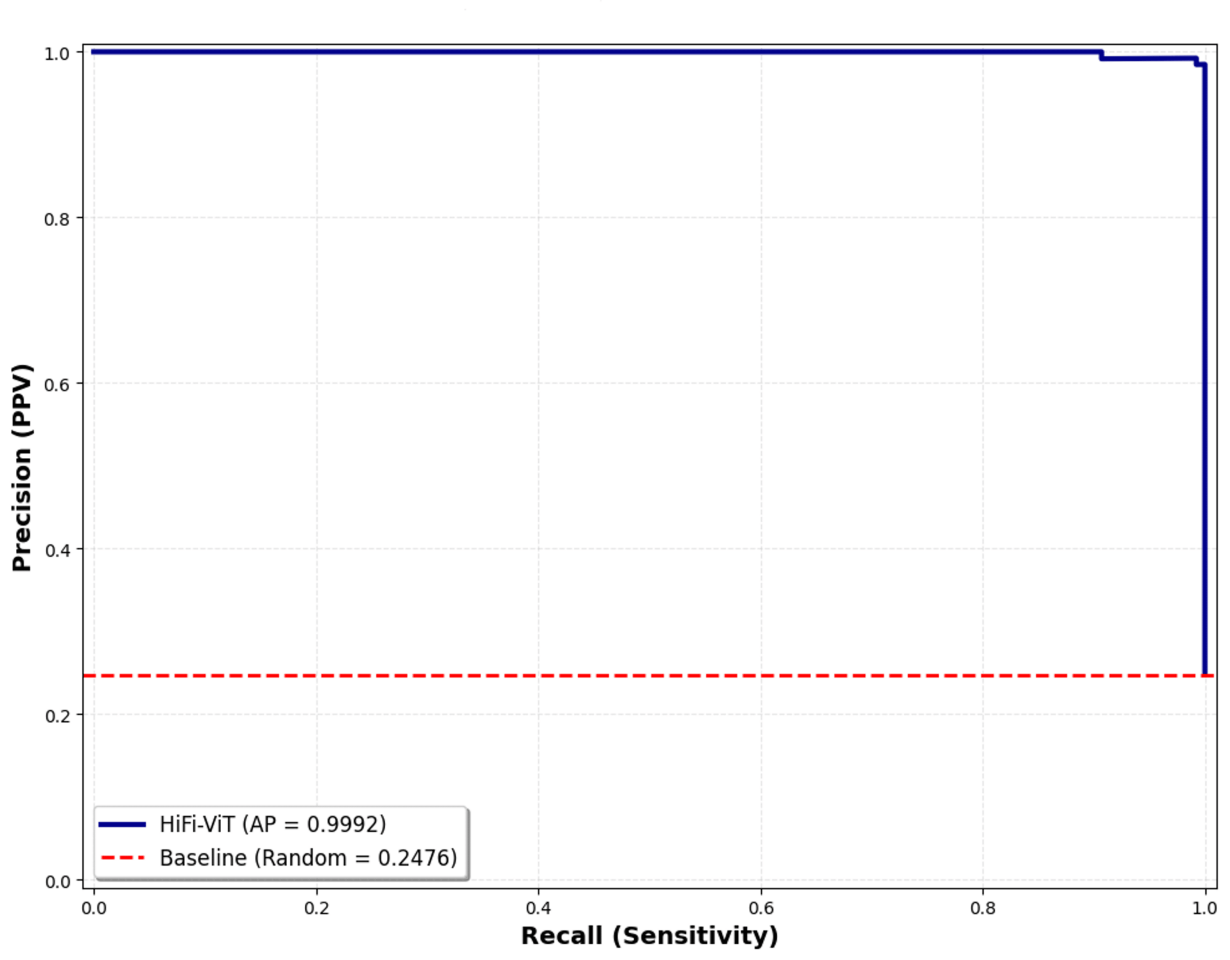
Precision-recall curve analysis for fibroid detection The ROC curve demonstrates excellent discrimination ability with an AUC of 0.9997, indicating strong separation between fibroid and normal classes. HiFi-ViT: Hierarchical Fibroid Vision Transformer; ROC: receiver operating characteristic; AUC: area under the curve

The precision-recall curve is specifically useful for evaluating binary models on imbalanced datasets, such that one class is much scarce than the other. It plots the positive predictive value (precision) against sensitivity (recall) at different thresholds (Figure 7). 

Average Precision: 0.9992

This summarizes the precision-recall curve and demonstrates the weighted mean of precisions achieved at each threshold, with the increase in recall from the initial threshold used as the weight. An average precision of 0.9992 is high, showing that the algorithm maintains extremely high precision similar to its recall. This means that the model is very good at detecting positive cases (fibroid) without getting many false positives. This is significant in medical diagnosis where both identifying actual cases (high recall) and minimizing incorrect diagnoses (high precision) are vital.

Baseline (Random Classifier) for This Dataset: 0.2476

The baseline shows the precision that would be achieved in a random model, which is the average case of positive cases in the dataset. This was achieved by dividing the number of fibroid samples by the total number of samples. The average precision of 0.9992 is extremely higher than 0.2476, which is the baseline, showing strong predictive power that is far beyond random chance. 

Evaluation of the model on raw ultrasound images

**Figure 8:**
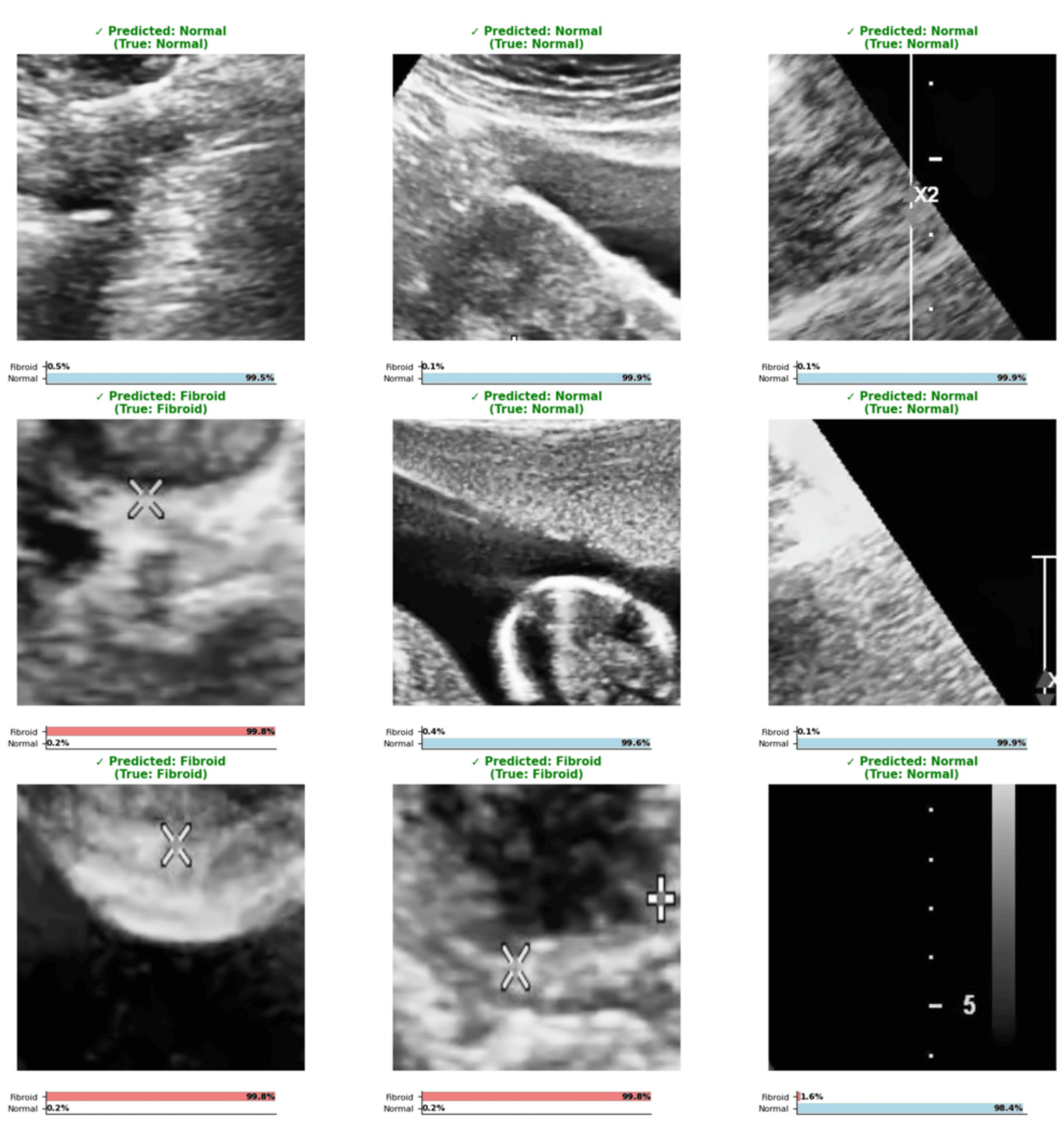
HiFi-ViT prediction results on raw validation images Representative predictions from the HiFi-ViT model on validation set ultrasound images, showing predicted class labels (fibroid/normal) with confidence scores. Images are from the publicly available uterine fibroid ultrasound dataset [17]. The model demonstrates high confidence in correct predictions, with fibroid cases showing >99% confidence and normal cases showing >98% confidence. HiFi-ViT: Hierarchical Fibroid Vision Transformer

The model was evaluated on randomly selected ultrasound images from the validated set, and the result showed that it correctly predicted these images with a very high probability for both classes (fibroid and normal) alongside the probability of prediction (Figure 8). 

Prediction of correct vs. incorrect classes

**Figure 9:**
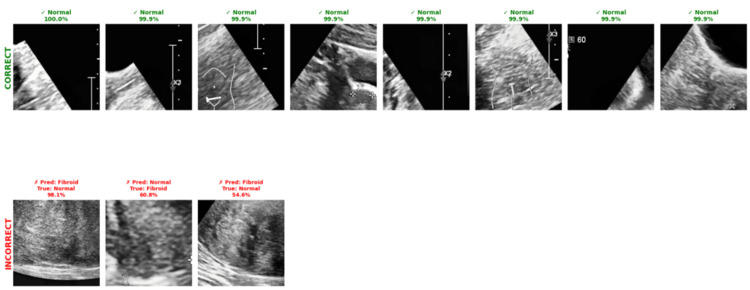
Correct vs. incorrect fibroid prediction analysis Examples of correct and incorrect classifications by the HiFi-ViT model on test images from the uterine fibroid ultrasound dataset [17]. Correct predictions (top row) show confidence scores ranging from 99.9% to 100%, while incorrect predictions (bottom row) show lower confidence scores between 54.6% and 98.1%, suggesting the model's uncertainty on challenging cases. HiFi-ViT: Hierarchical Fibroid Vision Transformer

The model's correct and incorrect predictions were explored with a higher percentage that ranges between 99.9% and 100% for correct classes, while incorrect classes range between 54.6% and 98.1% (Figure 9).

## Discussion

Table 3 presents a comparison of various methods along with their reported accuracy rates. For instance, the approach in Behboodi et al. [15] achieved 95.1% accuracy in classifying ultrasound images of the uterus, underscoring the strength of their method. Building on similar architectures, Dilna and Hemanth [16] applied UNet-based networks to ultrasound diagnostics and hit 86.2% accuracy. In a different vein, Behboodi et al. [15] applied deep learning to the ChEMBL dataset, reaching 85% accuracy and highlighting its potential. Meanwhile, Tang et al. [26] introduced the AR-Unet network, testing it on their custom dataset with a solid 94.56% accuracy, which speaks to the model's reliability. Yang et al. [27] further demonstrated the value of neural networks in medical imaging by processing ultrasound scans at 88.5% accuracy. Shifting to data mining, Girija and Varshney [28] analyzed records from 450 patients using multiple algorithms and obtained 89.54% accuracy, emphasizing its role in healthcare analytics. Lastly, Huo et al. [29] employed a deep learning model on 3870 ultrasound images, yielding 87.45% accuracy.

**Table 3:** Comparison of results with the proposed approach HiFi-ViT: Hierarchical Fibroid Vision Transformer

Author	Approach	Dataset	Accuracy measure
Behboodi et al. 2021 [15]	Classification	Ultrasound scanned uterus image	95.1%
Dilna and Hemanth 2018 [16]	UNet-based networks	US diagnostic imaging	86.2%
Li et al. 2022 [25]	Deep learning	ChEMBL database	85%
Tang et al. 2020 [26]	AR-Unet network	AR-Unet dataset	94.56%
Yang et al. 2023 [27]	Deep neural networks	Ultrasound image dataset	88.5%
Girija and Varshney 2022 [28]	Data mining techniques	450 patient datasets	89.54%
Huo et al. 2023 [29]	Deep learning techniques	3870 ultrasound image dataset	87.45%
Proposed methods	HiFi-ViT two-stage hierarchical approach	Ultrasound image dataset	99.42%

The HiFi-ViT framework stands out with its 99.42% accuracy, marking a clear step forward from these earlier works. This boost comes from a few key design choices. The two-stage setup tackles class imbalance head-on by extracting balanced patches of fibroid and normal tissue, ensuring fair training for classification. In the first stage, the DETR pinpoints fibroids accurately without relying on predefined anchors or post-processing steps like NMS. Then, the ensemble of three ViT in the second stage draws on self-attention to blend local details like textures with broader context, going beyond what standard CNNs typically capture. To provide preliminary clinical validation, three National Health Service (NHS) radiologists performed a qualitative review of a representative subset of ultrasound images with model-predicted bounding boxes. The radiologists examined whether the detected regions corresponded to anatomically plausible fibroid locations and confirmed that the DETR-generated bounding boxes were consistent with expected fibroid appearance and positioning. This expert review provided clinical face validity for the localization component of the system. However, a formal quantitative reader study comparing radiologist diagnostic performance with model predictions was not conducted and remains an important direction for future work.

From a clinical perspective, the proposed framework may support the earlier and more consistent identification of fibroids in ultrasound images. From a practical and service-delivery perspective, it may help reduce diagnostic workload, improve decision support, and enhance efficiency in imaging workflows.

In summary, these results showcase how machine learning and deep learning are advancing healthcare imaging, each method bringing unique strengths to the table.

Limitations

That said, this study is not without its limitations. The dataset of 871 ultrasound images is adequate for an initial proof-of-concept but remains relatively small compared to larger medical imaging datasets. More reliable results and stronger real-world applicability would likely be achieved with larger and more diverse datasets that capture variations in fibroid types, patient demographics, and ultrasound acquisition conditions.

Another limitation is that the publicly available dataset does not provide patient-level identifiers, meaning it cannot be confirmed whether each image represents a unique patient or whether multiple scans originate from the same individual. Although the dataset was divided at the image level prior to patch extraction to reduce the risk of direct data leakage, patient-level independence between training and test sets cannot be fully guaranteed. Additionally, the dataset contains only nine original normal ultrasound images, which required the use of patch extraction and augmentation strategies to balance the classes. While this approach improves training stability, it may limit the representation of the full variability of healthy uterine tissue.

Furthermore, the current framework performs binary classification (fibroid vs. non-fibroid) and does not distinguish between fibroid subtypes or quantify clinically relevant parameters such as fibroid size or volume, which are often important for treatment planning. Finally, the DETR-ViT pipeline requires substantial computational resources, such as GPU acceleration, to achieve efficient inference, which may limit deployment in low-resource clinical environments. Future work will focus on multi-site validation, real-time clinical evaluation, multimodal data integration, and model optimization for faster and more accessible deployment.

## Conclusions

Uterine fibroids are a gynecological condition that can significantly affect a woman's health and quality of life. Fibroids can prevent a fertilized egg from implanting or stop blood flow to the uterus, which can result in infertility or repeated miscarriages. Therefore, preserving fertility requires the early diagnosis of uterine fibroids. These hazards can be decreased, and women's chances of getting pregnant and carrying a child can be increased by treating fibroids early.

Even though not all fibroids have symptoms or need treatment, identifying those with a higher chance of causing problems can assist in tailoring treatment approaches to each patient's needs. This can reduce the effect of fibroids and the need for needless therapies. The absence of trustworthy uterine fibroid diagnostic tests is another difficulty; hence, there is a need for the proposed HiFi-ViT two-stage hierarchical approach that combines DETR with ensemble ViT for fibroid classification.

Future work will focus on validating the proposed framework on larger and more diverse clinical datasets and exploring its potential integration into real-world clinical decision support systems.
